# Atmospheric controls on the precipitation isotopes over the Andaman Islands, Bay of Bengal

**DOI:** 10.1038/srep19555

**Published:** 2016-01-25

**Authors:** S. Chakraborty, N. Sinha, R. Chattopadhyay, S. Sengupta, P. M. Mohan, A. Datye

**Affiliations:** 1 Indian Institute of Tropical Meteorology, Pune-411008, India; 2 Department of Ocean Studies and Marine Biology, Pondicherry University, Port Blair-744112, Andaman and Nicobar Islands, India

## Abstract

Isotopic analysis of precipitation over the Andaman Island, Bay of Bengal was carried out for the year 2012 and 2013 in order to study the atmospheric controls on rainwater isotopic variations. The oxygen and hydrogen isotopic compositions are typical of the tropical marine sites but show significant variations depending on the ocean-atmosphere conditions; maximum depletion was observed during the tropical cyclones. The isotopic composition of rainwater seems to be controlled by the dynamical nature of the moisture rather than the individual rain events. Precipitation isotopes undergo systematic depletions in response to the organized convection occurring over a large area and are modulated by the integrated effect of convective activities. Precipitation isotopes appear to be linked with the monsoon intraseasonal variability in addition to synoptic scale fluctuations. During the early to mid monsoon the amount effect arose primarily due to rain re-evaporation but in the later phase it was driven by moisture convergence rather than evaporation. Amount effect had distinct characteristics in these two years, which appeared to be modulated by the intraseasonal variability of monsoon. It is shown that the variable nature of amount effect limits our ability to reconstruct the past-monsoon rainfall variability on annual to sub-annual time scale.

Moisture generation and transportation is an important aspect of the hydrological cycle; this is especially important in the context of Indian monsoon as the agricultural and economic activities of the country significantly depend on summer monsoon rainfall. A large part of the Indian Ocean including the northern Indian Ocean produces and exports major amount of moistures to the Indian subcontinent resulting copious rainfall during the rainy season. The Bay of Bengal (BoB), an integral component of the Indian Ocean also contributes a significant amount of moistures resulting intense precipitation in the eastern parts of India and the neighboring countries. The BoB possesses certain unique characteristics which are believed to play an important role in moisture generation and transport process. Firstly, it maintains a relatively high SST especially during the monsoon season than the rest of the Indian Ocean which often exceeds 28 °C favouring convection^1^. Despite high SST and convective activities the BoB acts as a major sink of water vapor during the summer months (May to Sep) as precipitation (P) far exceeds the evaporation (E)^1^ but provides vapours to atmosphere during the rest of the year^2^. The Bay of Bengal strongly responds to Intra seasonal oscillation (ISO) and it is believed that it plays a crucial role in modulating this process. Its subsurface salinity and temperature structure often display distinct ISO signatures^3^. It is hypothesized that the air-sea fluxes of moisture and momentum influence the intra seasonal variability of the monsoon atmosphere^4^.

The Bay of Bengal is also characterized by low pressure systems (LPS) that cause extreme rainfall events especially in the coastal regions of India, Bangladesh, Myanmar, Sri Lanka etc. Hence identification of moisture sources consists of an important aspect of the hydrological study which can address issues like understanding the monsoon variability, water security, managing and mitigating the extreme rainfall events caused by tropical cyclone etc. Apart from this it is also useful in paleoclimatic reconstruction and future climate change scenario.

Identifying source and sink of atmospheric moistures are done mainly by numerical means, such as box model calculation, and numerical water tracer analysis^2^,^5^. A third approach is the physical tracer analysis; that is, the study of stable isotopes in precipitation which is considered a reliable means in obtaining source related information^6^. However despite it being a powerful tool there are some issues, such as, the relationship between rainfall isotope values and precipitation sources need to be addressed as the isotopic fractionation during air mass transport may alter source signature^7^. In this regard it is crucial to improve our understanding of how moisture sources, convective activities affect precipitation isotopes, which will also help in interpreting the paleoclimatic archives, such as tree rings, ice cores, cave deposits etc^2^.

The Andaman Islands is a group of island strategically located in the Bay of Bengal as far as monsoon circulation is concerned. Being an island and with a large forest cover (~ 92%) the secondary source of moisture (i.e. soil moisture) is expected to be low and hence the isotopic study of its rain water is likely to provide valuable input in understanding the moisture dynamical processes. But no systematic study of isotopic analysis of rain water in this region is available, except a short time series (25 May–31 July, 2010) of δ^18^O of Port Blair rain^8^,^9^. Hence the characteristics behavior of rainwater isotopes largely remains unknown, especially on short time scales. We have undertaken a systematic study to decipher the isotopic characteristics of rain water in this region as part of an IAEA project entitled ‘Stable Isotopes in Precipitation and Paleoclimatic Archives in Tropical Areas to Improve Regional Hydrological and Climatic Impact Models’ initiated in 2013. The isotopic records of Port Blair rain for the year 2012 and 2013 are presented. The main objective is to examine the nature of the precipitation isotopes in this region, how they are controlled by the ocean-atmosphere variabilities, such as, moisture source and transport, convective activities etc. What is the nature of the amount effect and if it is controlled by the intra seasonal variability of monsoon. Whether the rain water isotopic composition is controlled by rainfall events or by the moisture fluxes that is believed to influence the intraseasonal characteristics of monsoon^4^.

## Study area

**Figure 1:**
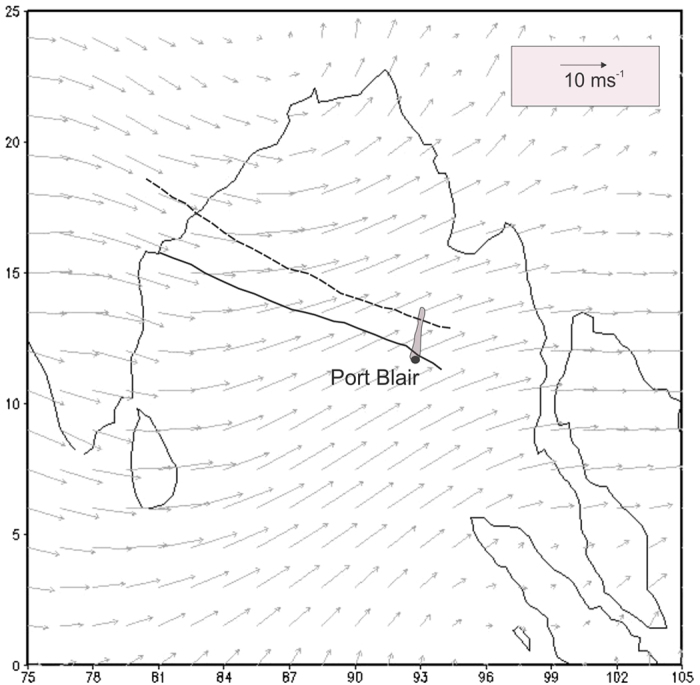
Sample location and cyclonic storm track. The Andaman and Nicobar Islands (in grey, not to scale); Port Blair (solid circle) is the rain sampling location. The two curve lines show the cyclonic tracks: *Phailin* (dotted) and *Lehar* (solid) that caused heavy rain in and around the Islands in Nov-2013. According to the IMD report the Phailin originated from a remnant cyclonic circulation from the South China Sea which turned to a depression over north Andaman Sea on 8^th^ Oct 2013 near 12 °N 96 °E. On the other hand the Lehar originated over the Andaman Sea on 24^th^ Nov 2013 near latitude 10 °N and longitude 95 °E. The mean wind pattern during the monsoon season (JJAS) of 2012 has also been shown (grey arrows). Vector plot was created by open software GrADS (http://iges.org/grads/) and superposition of storm tracks were done by licensed CorelDRAW.

The Andaman and Nicobar Islands are a group of islands located in the south east portion of the Bay of Bengal (10.5–13.5 °N, 92.5–93 °E) with a north-south trend (Fig. 1). The island receives heavy rain during the summer monsoon which extends from mid May to early October. Mean annual (area averaged) rainfall is about 3180 mm, which is more than three times than that of the Indian Summer Monsoon Rainfall (ISMR ~ 890 mm; India Meteorological Department; IMD). About 90% (10%) of the annual rainfall takes place during summer (winter). The total geographical area is about 8250 sq km, most of which (~92.2%) is covered by forest. The seasonal variations of climatological mean rainfall and temperature for the Port Blair region are shown in supplementary Fig. S1.

## Results

**Figure 2:**
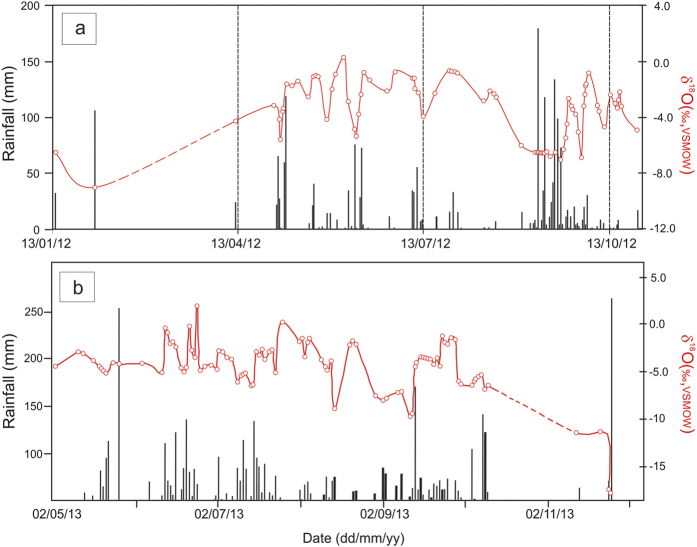
Time series of daily rainfall and δ^18^O. The black bars represent raingauge data (Port Blair) and red line is the corresponding δ^18^O record. The upper panel (**a**) represents the year 2012 and the lower panel (**b**) is for 2013. An inverse correlation between rainfall and δ^18^O record is apparent for 2012. Plots have been created using licensed Microsoft Office (Excel).

Figure 2 displays the oxygen isotope time series for the year 2012 (upper panel) and 2013 (lower panel). The typical values of minima and maxima of δ^18^O (δD) are approximately −7 and 0% (−50 and +6%, relative to VSMOW) for summer monsoon of 2012. Due to paucity of data the winter season is represented only by a few samples.

The monsoon onset in the Andaman area usually takes place on 20^th^ May^10^, though pre-monsoon showers begin in early May. The oxygen isotopic values during the early season had a narrow range (−1 to −4%) with occasional depletions until late August. Henceforth, high depletion was observed which continued till the first week of September. The δ^18^O became less than −6% and δD recorded below −40%. The low values persisted for several days and in the middle of September the δ^18^O again increased but remained somewhat lower than the mean value registered in the months of MJJ.

The year 2013 showed somewhat different behavior. Unlike 2012 the May-June isotopic record in 2013 did not show any depletion, rather pulses of positive values were observed in June. Positive values could arise due to re-evaporation of rain drops; it is also apparent from a strong anti-correlation between δ^18^O – d-excess (discussed later). Depleted isotopic values began to occur in July and like 2012 the September 2013 rainfall also showed significant negative excursion. In this year, the δ^18^O had a little higher amplitude (0 to −8%) compared to 2012. In both the year the early monsoon (May) showed a mean δ^18^O ca. −2 to −3%. These are the typical values observed in tropical islands^11^. Our results also match for the comparable time period with that of the δ^18^O of rainfall reported in Laskar *et al.*^8^ who measured oxygen isotopic values of Port Blair rainwater from 25/5/2010 to 31/7/2010. But the isotopic values could be modulated by large scale processes, such as organized convection associated with the monsoonal intra-seasonal oscillation^12^. Additionally, low pressure systems can significantly reduce the rain water isotopic compositions^13^.

### Local Meteoric Water Line

The local meteoric water lines for the years 2012 and 2013 are given below. The scatter diagrams (δ^18^O vs. δD) have been shown in Fig. S2.


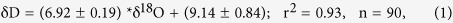



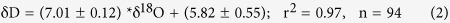


Both the slope and the intercept are lower than that of the global meteoric water line (GMWL; 8 and 10 respectively). Lower values of slope and intercept than the GMWL often indicate the presence of secondary moisture source^14^. Since the Andaman Islands is relatively small and has little fresh water deposit the secondary source of moisture can mostly originate by rain water re-evaporation. The slope remains the same within the limits of uncertainty level for both the year asserting that overall time averaged, moisture recycling process, more or less, remained the same. But the intercept differed considerably in 2012 and 2013, probably representing a variable vapour source contributing significant amount of rainfall in this region.

### Deuterium excess

**Figure 3:**
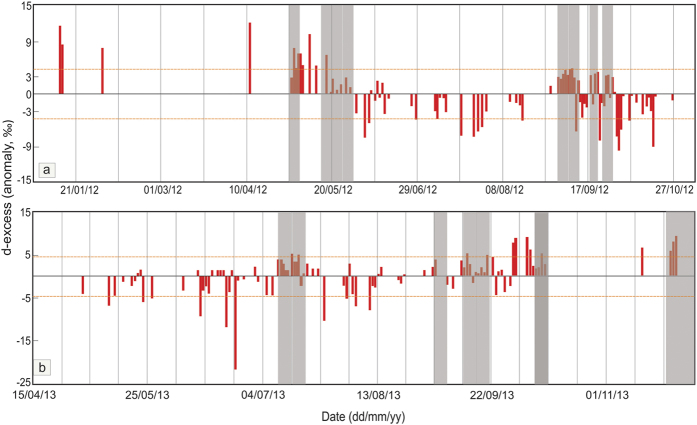
Time series of d-excess anomaly. (**a**) d-excess anomaly of daily rainfall for year 2012 and (**b**) year 2013. The anomaly shows distinct seasonality; positive anomaly occurs mainly during the winter/spring and negative anomaly during the monsoon season. The isolated events of positive d-excess anomalies during the monsoon season are caused by large scale organized convection. Positive anomaly during November 2013 coincided with the heavy cyclonic activities. Orange lines represent ±1σ variability. Graphics were made using licensed Microsoft Office (Excel) and CorelDRAW.

The deuterium excess ranges from 4 to 25% in year 2012 and −12 to 18% in year 2013. We have calculated d-excess anomaly by subtracting the seasonal mean from the individual values. Figure 3a,b show this anomaly for the respective years. The winter/spring and pre-monsoon times are characterized by positive anomaly while the monsoon season typically shows negative anomaly. This is the typical nature of d-excess variability found in the northern hemisphere on monthly/weekly time scale, including the South Asian^15^ and Indian^16^ region. However, the September month in both the year is characterized by positive anomaly when the precipitation isotopes suffered from high depletions. On the other hand, positive anomaly was observed in Nov-2013 when severe cyclonic activities were reported. One important aspect is that positive d-excess anomalies are usually associated with large scale organized convection during the summer monsoon season. The shaded regions of Fig. 3 represent the convective activities (based on pentad OLR anomaly) when d-excess displayed higher values.

### Isotopic variability associated with low pressure systems

The tropical cyclones (TC) are known to produce most depleted isotopic values^13^. The isotopic analysis of precipitation during cyclonic events in the Bay of Bengal, to our knowledge, has not been systematically studied. This work, however, does not aim to do so but preliminary analysis shows that precipitation isotopes underwent a varying degree of isotopic fractionation during heavy to severe cyclonic storms that occurred during the latter part of the year 2013. A qualitative analysis is presented.

In the first week of October-2013 a low pressure system originated east of the Andaman Island causing heavy rainfall: ~55 mm on 4^th^ Oct at Port Blair. The following three days there was little rain but heavy rain (91 mm) was recorded on 8^th^ Oct at Port Blair. The δ^18^O (δD) of precipitation during all these days had a close range of −4.9 ± 0.60% (−25.87 ± 3.8). The same day the depression intensified and eventually turned into a Super Heavy Cyclonic Storm (near 12 °N, 96 °E, the “*Phailin”*). The following day it crossed Andaman Islands and moved northwards^17^. On 9^th^ and 10^th^ Oct the recorded rainfall at Port Blair was ca. 73 and 10mm and the corresponding δ^18^O was −5.5% on these days. The inset in Fig. 1 shows the storm tracks; the dotted line is that of *Phailin*. The cyclonic effect soon resumed and the Port Blair site recorded moderate rain on 13^th^ Nov (13.8 mm). There was little rain on 23^rd^ Nov but the δ^18^O showed nearly the same values (ca. −10.4%) on both these days. Mean time, another depression formed south east of Andaman island around 19 Nov 2013 (approximate location 10 °N, 95 °E)^17^. When it reached the island it turned to Cyclonic Storm (the *Lehar,* the continuous line in Fig. 1) and caused heavy rain in the southern parts of the Andaman Island. The recorded rainfall on 24^th^ and 25^th^ Nov were 21 and 213 mm respectively (Fig. 2b). Interestingly, the isotopic values observed during these two days were nearly the same, δ^18^O ~ −17% (δD ~ −118%) despite there was one order of magnitude difference in rainfall amount.

It appears that the isotopic data do not show linear behavior with the rain amount during the cyclonic storms. During the first few days of *Phailin* the rainfall showed a wide variability; from near zero to 91 mm, but the δ^18^O remained nearly constant at −4.6 ± 0.44%. During the *Lehar* cyclonic activity rainfall again showed a large variability- from near zero (on 23^rd^ Nov) to over 200 mm (on 25^th^ Nov). But isotopic composition of rainfall remained nearly the same (δ^18^O ~ −17 and δD ~ −118%). It also implies that the isotopic values of rain depend more on the available moisture and its isotopic composition rather than the rain amount, but in this process it carries ‘memory’ of the rainfall (discussed later).

This is also to be noted here that the isotopic values displayed linear dependency to the distance of the cyclonic events. The *Phailin* track was relatively far from the rain sampling location (~160 km), compared to that of *Lehar* (~25 km). Hence the most depleted δ^18^O (δD) values recorded during *Phailin* was −5.8% (−31.84%), but the same for *Lehar* was −17.1% (−118.5%).

## Discussion

**Figure 4:**
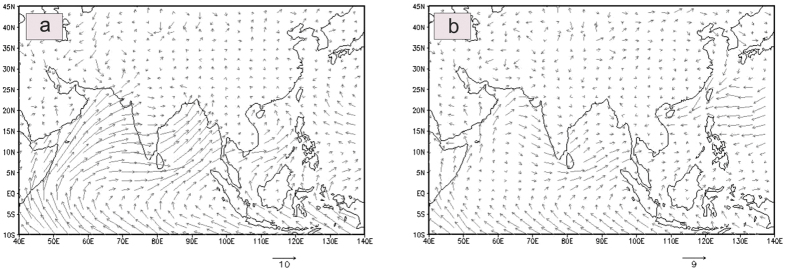
(**a,b**) Moisture flux vector over northern Indian Ocean. Moisture flux (g.kg^−1^.m.s^−1^) has been calculated based on two sets of dates. First set consists of those dates when normalized d-excess anomaly <−1 (left panel) and the second set consists of dates when normalized d-excess anomaly >+1 (right panel). The first set almost exclusively belongs to the summer monsoon season, while the second set comprises the non-monsoon period as well as limited time bands during the monsoon season when d-excess showed high values, as shown in shaded time zones in Figure 3. Maps were created by open software GrADS (http://iges.org/grads/).

The seasonal variations of d-excess (shown as anomaly in Fig. 3) could be explained by means of moisture source. Firstly we categories d-excess data in two sets. For this purpose d-excess has been normalized by subtracting the mean value from the individual value and then dividing by its standard deviation. The first (second) data set consists of normalized d-excess <−1 (>+1). These two data sets yield two sets of dates in which normalized d-excess is either <−1 or >+1. These two sets of dates have been shown in Table ST1 (supplementary section). Moisture flux vector was calculated over an area (10 °S–45 °N, 40 °E–140 °E) for the corresponding sets of dates yielding (normalized) d-excess <−1 and >+1 respectively. Figure 4a,b clearly shows strong (weak) moisture flux in the case of normalized d-excess <−1 (>+1). To demonstrate further we have taken three subsets of arbitrary dates (04/06/2012, 25/07/2013, 10/08/2013) belonging to monsoon (i.e., d-excess <−1) and three non-monsoon dates (viz. 13/01/2012, 11/04/2012, 23/11/2013) (i.e., d-excess >+1). 98 hr back trajectories using HYSPLIT model have been calculated on these dates to trace the moisture source. Figure ST1 (supplementary materials) clearly shows that in case of the first (second) set, which is characterized by d-excess <−1 (>+1) moisture was mainly sourced from the equatorial Indian Ocean (continental/non-Indian Ocean) region. This demonstrates that low d-excess values typically seen during the monsoon season arise due to moistures coming from the equatorial area across the Indian Ocean. But the moistures during non-monsoon time are characterized by higher d-excess and usually originate in continental and/or non-Indian Ocean region.

On monthly time scale d-excess also varies. The positive d-excess anomaly in early May is due to a deep convection originating over a wide area in south Bay of Bengal including the Andaman Islands as shown in pentad OLR anomaly for May 1–5, 2012 (Fig. S4); 24 hr back trajectory analysis reveals that moisture was sourced from the neighboring oceanic region, ca. 9 °N, 89 °E (Fig. S4) which produced higher d-excess rain in Port Blair. Similarly, the positive anomalies observed during the latter part of May-2012, and July-2013 were also due to varying degree of convective activities as revealed in the pentad OLR anomalies (Fig. S5, July-2013 anomaly not shown). Recycling of water vapor within the convective cell makes the rainfall low in isotopic values but high in d-excess resulting an inverse correlation between these two parameters^18^. Such kind of correlation between OLR and d-excess has also been reported in the tropical region^19^,^20^.

On the other hand non-convective processes result relatively higher δ^18^O and lower d-excess in precipitation^21^. The negative d-excess anomaly during JJAS suggests that the humidity of the ocean surface is relatively higher than the average relative humidity (RH) of 81% observed in tropical region^22^ and secondly the major moisture source is the remote oceans^23^. This is corroborated by 98 hr back trajectory to the sampling site derived from Hysplit model which shows (Fig. S6) that the moisture source was mainly confined to the equatorial Indian Ocean region during the JJAS season.

The isotopic compositions of rainwater in the tropical region typically demonstrate an anti-correlation with the rainfall amount, known as ‘amount effect’ which shows a varying pattern in the Indian subcontinent. While most of the places obey this relation, some places such as, north-east India, west coast of India and parts of south India show notable exceptions^16^,^24^,^25^,^26^. Since amount effect is related to various atmospheric factors, such as, rainwater re-evaporation^19^,^27^, the degree of organized convection^13^,^28^, moisture flux convergence^29^ etc., it is important to examine which processes are operative in the Andaman region.

Figure 2a shows an apparent inverse correlation between rainfall amount and its oxygen isotopic composition yielding a low correlation, r = −0.29 (n = 90, p = 0.002; Fig. S3a). But the year 2013 shows somewhat different behaviour. As obvious from Fig. 2b the δ^18^O depletions are not systematically correlated with the increased rainfall amount. Though the regression analysis on daily time scale for all the individual cases (r  = −0.29, n = 94, p =  0.002) yields a similar value to that of year 2012, the relation is almost lost (see Fig. S3b) when the monsoon months (JJAS) are considered. For JJAS the correlation coefficients are −0.29 and −0.083 for the year 2012 and 2013 respectively. Similarly, when the daily (interpolated) JJAS δ^18^O values are correlated with the TRMM derived rainfall data the correlation coefficients for the year 2012 is reasonable (−0.24) but vanishes for the year 2013 (r = 0.02). The relationship is improved only when the rainfall and rain δ^18^O are averaged on monthly scale, being −0.73 and −0.59 for 2012 and 2013 respectively. The above observations clearly indicate that amount effect is differently manifested in these two years.

**Figure 5:**
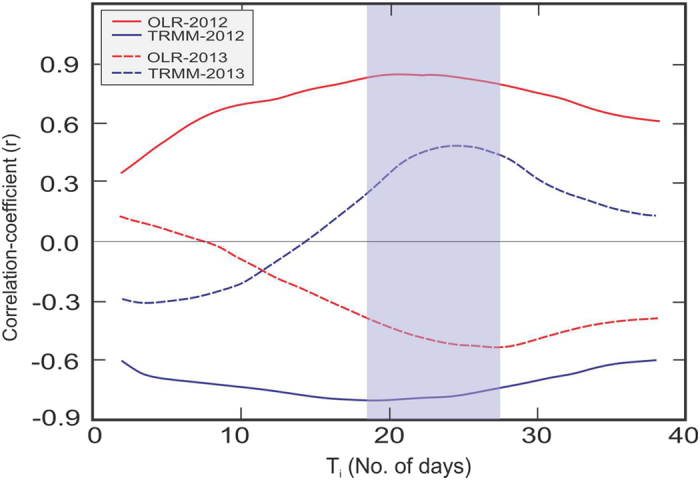
Correlation diagram for δ^18^O vs. convective parameters. The progress of correlation between δ^18^O and the convective parameters (TRMM derived rainfall and OLR) integrated over a time period T_i_. The rainfall and OLR data have been integrated over a grid size of 90°–95 °E and 7.5°–12.5 °N centered at Port Blair and integrated for T_i_ days preceding the event. The blue (red) curves represent the rainfall (OLR). The solid (dashed) lines are for the year 2012 (2013). The maximum correlation (p = 0.001) is obtained when rain (OLR) data have been integrated for a period of 18–21 days for the year 2012 and 24–27 days for 2013 respectively. Note that a normal amount effect [viz., δ^18^O = *f* (1/rainfall)] is present in case of year 2012 but the year 2013 shows a different behaviour. However both the years show a time band of about 18–27 days (shaded) in which the (amount) effect is maximum (see text for details). Plot was made using licensed Microsoft Office-Excel and CorelDRAW.

We have investigated the effect of convective activities on rainwater δ^18^O, similar to studies undertaken elsewhere^19^,^30^. δ^18^O of rain has been correlated with the TRMM derived rainfall (and OLR) integrated for T_i_ days preceding the event. Figure 5a shows the pattern of correlation coefficient with increasing values of T_i_. The analysis shows that on short time scale the correlation is low either with rain or with OLR, but improves when rain (also OLR) is integrated for several days. The maximum correlation is obtained for T_i_ =  18 (21) days. The year 2013 also shows similar characteristics, but the time band is 24–27 days (Fig. 5b). Risi *et al.*^19^ provided an explanation of such kind of behaviour in the context of rain isotopic analysis in Niamey, Niger. Each convective event causes depletion a little more to the low level vapor; the isotopic composition feeding a convective system is thus influenced by the cumulative effect of the previous convective systems. This means that a minimum time frame is required for rainwater δ^18^O in order to respond strongly to the convective systems, which in the case of Bay of Bengal is about 20–27 days. This also explains why the amount effect is poor on daily scale, but gets improved on longer time scale (i.e., >3 weeks). The improved correlation between δ^18^O - rainfall on higher temporal domain, implies that the isotopic properties of rainfall are controlled more by the integrated effect rather than the discrete events of rainfall. In other words, the isotopic tracers carry a ‘memory’ of the past rain^19^,^30^,^31^. This kind of memory effect has also been observed in case of rainfall during severe cyclonic events, as mentioned earlier. Also it means that when the individual local convective events become an organized convection the isotopic composition of rain water responds better to this large scale system rather than the isolated events. Such kind of observations have also been reported especially in the tropical region whereby precipitation isotope fractionation processes have been shown to integrate across wider spatial scales and longer time periods^28^,^30^,^31^,^32^,^33^.

**Figure 6:**
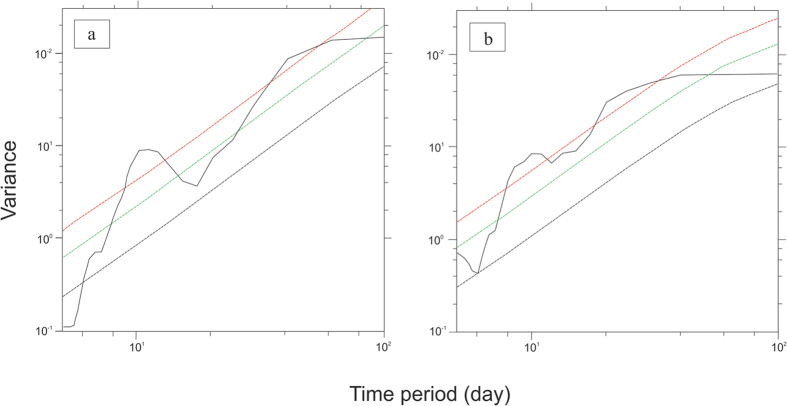
(**a,b**) Power spectrum of rainwater δ^18^O. Spectral peaks calculated for daily interpolated δ^18^O of summer rainfall (15 May to 15 Oct) of Port Blair for the year 2012 (**a**) and year 2013 (**b**). Year 2012 shows a significant peak at about 13 days and year 2013 shows strong peaks at 11 and 22 days. The colored lines are the mean red noise (green) and the upper (red, 95%) and lower (black, 5%) confidence limits. Anything above the red line is significant. Plots were made using open software NCL (http://dx.doi.org/10.5065/D6WD3XH5).

It is well known that Indian monsoon is characterized by two distinct modes of variability on intra seasonal time scale with broad spectrum peaks, viz., 10–20 day and 30–60 day^34^, that is stronger than synoptic scale variability. In order to examine if the rainwater δ^18^O is influenced by any of these modes we have done power spectrum analysis of daily δ^18^O (interpolated) data for the JJAS season, which shows statistically significant peak above red noise (as green curve with upper (95%) and lower (5%) confidence limits of the red noise marked by red and black curves respectively) at about 13 days (for 2012; see Fig. 6a) and 27 days (for 2013, Fig. 6b) respectively; this time band (13–27 day) matches quite well with the time scale obtained by correlation analysis. The 18–27 day timescale observed by correlation analysis appears to be linked to the monsoon intraseasonal oscillation that shows spectral signature on this time frame during monsoon season over the Indian subcontinent^34^ implying that rain δ^18^O is a likely recorder of ISO variability.

**Figure 7:**
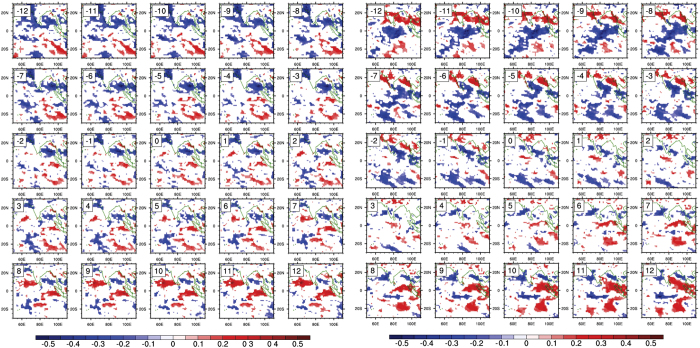
Lag correlation plot between rainfall and δ^18^O. Spatial correlation coefficient between δ^18^O (reference) and TRMM derived daily rainfall (sliding) data. In year 2012 (left panel) δ^18^O shows good negative correlation with rainfall amount at lag − 12 day which slowly weakens and nearly vanishes at lag + 12 representing approximately 24–25 day cycle of the evolution of the amount effect. Right panel represents the year 2013. Unlike 2012 where the negative correlation is visible mainly in Bay of Bengal in this year the negative correlation manifests in the Indian Ocean. Plots were made using free software NCL (http://dx.doi.org/10.5065/D6WD3XH5).

We have further investigated the nature of the δ^18^O -rainfall relationship by analyzing their spatial correlation pattern in order to understand the spatial variability and also the variation of amount effect in these two years. Figure 7a shows the lag correlation spatial diagram with δ^18^O data as the reference (fixed) time series and TRMM daily rainfall data at each grid point over the Indian Ocean region for the year 2012 during JJAS as the other (sliding) time series. The lag correlation plot showing sequential development of correlation pattern at each grid from lag − 12 to lag + 12 is shown here in separate panels.

Strong inverse correlation was observed in 2012 at lag − 12 days. The correlation progressively weakens and vanishes at lag + 12 days. This behavior essentially shows a sinusoidal behavior of amount effect with a periodicity of about 24–25 days. This time frame matches with that determined through correlation analysis and the spectral characteristics of δ^18^O showing influence of monsoon intra seasonal characteristics. Additionally, the correlation band (the region where correlation is negative) is restricted to northern Indian Ocean (Arabian Sea and Bay of Bengal); but negligible or weakly positive in the Indian Ocean.

The year 2013 shows similar cycle but the usual negative δ^18^O -rainfall correlation band shifts from the north Indian Ocean to equatorial IO (see Fig. 7b). Whereas the northern Indian Ocean region, unlike the year 2012 displays positive correlation. This demonstrates that the expected rainfall- δ^18^O negative correlation or the amount effect has a spatial variability; as evident in Fig. 7, the spatial extent is >1000 km. This kind of variability may arise due to the seasonality (i.e. season to season variation) of the monsoon intraseasonal oscillation^35^. The seasonality of monsoon ISO is linked to monsoon interannual variability^35^. For example, the year 2012 is characterized by relatively low rainfall^36^ (ISMR: 93% relative to long period average, LPA), positive SST anomaly in distinct areas of the Indian Ocean during the JJAS period representing relatively restricted moisture source regions. On the other hand, the year 2013 experienced higher than normal rainfall^17^ (ISMR:106% relative to LPA), strong intra-seasonal variability, the fastest spread of monsoon in India in the last 41 years, neutral SST anomaly from most parts of the Indian Ocean representing diverse moisture source areas^17^. It may be mentioned here that positive amount effect has also been reported in Niamey, Niger^19^, and in western India^24^. In this context it may be clarified why δ^18^O (at t = T) has been compared with future rainfall events (i.e. at t = T+n, where n is a positive number). Moisture can be generated through localised convective systems. It can also be generated by large scale organized convection which evolves with time. A priory, it is not known which system is responsible for producing the rain that is being observed/isotopically analyzed. Since a large scale system slowly evolves with time, the correlation would decay slowly with time as compared to the localised convective activity with short life span where correlation falls fast with lead-time. Hence in order to study the temporal behavior of the large scale system vis-à-vis its isotopic characteristics lead-lag correlation analysis has been performed.

The relation between RH and d-excess has also been used to assess the effect of RH on rainwater evaporation as raindrop evaporation results an inverse correlation between δ^18^O and d-excess^37^. We have observed strong correlation between δ^18^O (original values) and d-excess in early monsoon (for example, r^2^ = 0.35, (n = 10; p = 0.1) and 0.78 (n = 17; p = 0.001) in June 2012 and 2013 respectively). Though the RH during the early monsoon (May-June) remains relatively lower than the later part of monsoon (in 2012; see Fig. S7a), but the lack of similar behavior in the year 2013 (Fig. S7b) indicates that consideration of humidity alone may not fully explain the observed relation between δ^18^O and d-excess. The early phase of monsoon is characterised by strong change in circulation pattern. For example, in 2012 wind speed shows sharp rise- from about 6 ms^−1^ in mid May to more than 19 ms^−1^ in mid June (Fig. S7a). In the year 2013 the wind speed ranges from <6 ms^−1^ to about 17 ms^−1^ (mid May to 3^rd^ week of June; Fig. S7b). Since strong wind favours raindrop evaporation, δ^18^O - d-excess show strong inverse correlation in early monsoon. After the middle of June wind speed shows a gentle decreasing trend in both the years. It may also be noted that in the early phase of monsoon, wind speed and RH nearly co-vary but after June their variations are somewhat out of phase (Fig. S7). This means, relatively higher humidity and lower wind strength after June result a weak δ^18^O - d-excess correlation, as is evidenced from the r^2^ value which reduces to about 0.18 in Sep (both years).

Apart from δ^18^O – d-excess, we have also observed a modest correlation between d-excess and RH. A strong positive correlation between these two parameters emerges when rain drops evaporate significantly^38^. For the months of May to Jul, r^2^ for RH vs. d-excess linear fit is 0.2. With the progress of monsoon the environmental conditions change (relatively higher RH and lower wind speed, see Fig. S7) the relation between d-excess and RH weakens. The above observations imply re-evaporation of raindrops, to some extent is present especially during the early to mid monsoon season, which also contributes to amount effect^27^. This is also consistent with our earlier observation that the evaporated vapour (hence low in δ^18^O) is fed into the convective system and gets influenced by earlier convective events^19^, which essentially causes amount effect operating efficiently within a time frame of 18–27 days.

**Figure 8:**
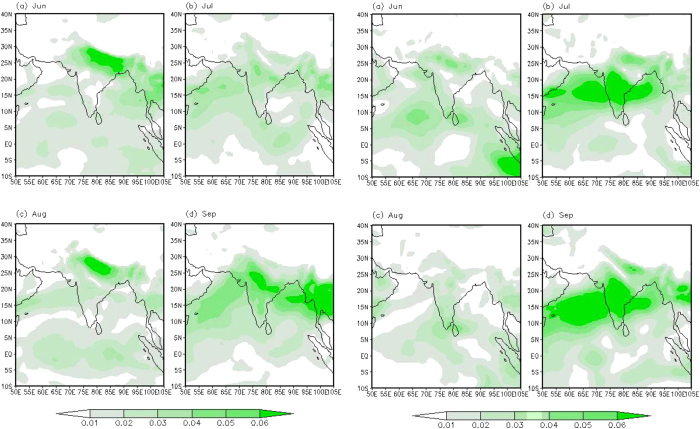
Moisture convergence showing relative variation on monthly scale. Moisture convergence calculated using specific humidity and zonal and meridional wind and plotted as anomaly for the Jun-Sep time period. Each panel (left and right) shows the monthly anomaly relative to the JJAS mean for the year 2012 (left panel) and 2013 (right panel) respectively. Maps were created by open access software GrADS (http://iges.org/grads/).

As mentioned earlier due to the changes in environmental conditions (determined mainly by RH and wind speed) during the later phase of monsoon the correlation between d-excess and RH weakens. For the month of September the correlation nearly vanishes (r^2^ = 0.0002, n = 25). Such kind of situation may be responsible in producing high and persistent depletion of isotopic records, observed more or less in both the years during September (Fig. 2) resulting an amount effect that could be explained by the hypothesis proposed by Moore *et al.*^29^. According to these authors when precipitation exceeds evaporation in a given region that is convectively active, amount effect is largely a result of isotopically depleted vapor converging in the lower and middle troposphere with smaller contributions from surface evaporation. Bay of Bengal in general is characterized by P/E >1 during the monsoon season^1^, which is more pronounced in September in the studied years. We have estimated the moisture convergence (see the Method section) for the JJAS season. Figure 8a,b show the anomaly (relative to the JJAS mean for each year) for the individual months showing the relative difference of moisture convergence during the monsoon season. As evident from these figures the northern Bay of Bengal experienced higher moisture convergence during September, asserting the Moore *et al.*^29^ hypothesis. 98 hours back trajectory calculations also show (Fig. S8) that moistures were originated mainly in the equatorial Indian Ocean region during September in both the years. However this Figure (8b) also shows significant amount of moisture convergence during July-2013, implying that this month also experienced similar characteristics as September. The δ^18^O time series (Fig. 2b) for 2013 indeed shows such kind of behavior, that is very similar δ^18^O *pattern* in these two months albeit with reduced amplitude in July-2013. Like Sep-2013, positive d-excess anomaly in July-2013 also provides supportive evidence.

## Conclusions

The rainwater isotopic compositions over southern Bay of Bengal show systematic depletions which seem to vary with the seasonal cycle of monsoon as well as seasonality of the intraseasonal oscillation. Relatively low humid condition but strong circulation in the early phase of monsoon cause moderate level of raindrop re-evaporation, which in turn contributes to amount effect. However the extent of evaporation diminishes, caused by altered atmospheric condition at the mature phase of monsoon when amount effect seems to be controlled more by the moisture flux convergence than the local evaporation.

On seasonal time scale the d-excess shows lower values during the monsoon but higher values during the non monsoon time. This appears to be controlled by the moisture source, originated in the equatorial Indian Ocean during monsoon but from continental and non-Indian Ocean region during the non-monsoon time; this has also been confirmed by back trajectory analysis. However, higher values of d-excess could also arise as isolated events during the monsoon which is caused by re-cycling of water vapour being systematically fed into a large scale convective system. The time scale of the positive d-excess anomalies during the monsoon appears to be modulated by the monsoon intra-seasonal oscillation.

Convective activities integrated over a period of time control rainwater isotopic composition more than the isolated events. Hence the rain δ^18^O carries the signature of previous rainfall better than the individual rain events. The integrative characteristics of rain isotope and in turn the amount effect manifest the best in a time scale of 3–4 weeks that appear to be linked to the monsoon intra seasonal variability which is a very specific characteristics of the Bay of Bengal.

Since the amount effect responds to integrated effect of convective activities it depends basically on mean state of the monsoon operating on intra seasonal time scale; that is, spatially over 1000 km and temporarily approximately 12–27 days. The monsoon in 2012 and 2013 underwent different intra seasonal variability and hence amount effect also showed very different behavior in these two years. This is an intricate attribute of the amount effect which is likely to have important implications in paleo-monsoon reconstruction. According to current understanding, amount effect implies- more the rain, more is the isotopic depletion. But this study shows that increased amount of rainfall does not necessarily mean a higher depletion in ^18^O or D. Since amount effect is caused by different atmospheric processes which occur in different scales, their interactions introduce non-linearity resulting a variable amount effect. Hence this characteristic behavior of amount effect is a limiting factor for paleo-monsoon reconstruction on annual to sub annual time scale using speleothem or tree ring.

Currently the spatial pattern of ISM rainfall and its isotopic variability simulated by global circulation models show good agreement with the observations, but large discrepancies exist in the magnitude of simulated amount effect^39^. Our study is expected to provide a better understanding of the amount effect in the Indian region which in turn is likely to help in reducing the discrepancy between observation and the model simulation.

### Data and Methods

Daily rain water was collected from the Pondicherry University Campus (11.66 °N, 92.73 °E; 11 pm, local time) at Port Blair. About 90 samples were collected in each year. The month of September received near continuous rainfall (29 and 24 days in 2012 and 2013 respectively). The rainwater was collected in a 5 lt carbuoy following a procedure similar to Deshpande *et al.*^39^,^40^. The samples were transferred to leak proof plastic bottle and then shipped to the Indian Institute of Tropical Meteorology (IITM), Pune. Initially the samples were analyzed using a Thermo Scientific Delta V Plus isotopic ratio mass spectrometer (IRMS) and later by an LGR Water and Water Vapor Analyzer (Model: TIWA-45-EP). All samples including the previously measured samples by the IRMS were re-analyzed for δD and δ^18^O. The isotopic ratios measured using both the machines agreed within the ±1σ variability. A few samples that did not match within the ±1σ variability were discarded. The overall analytical precision obtained for δ^18^O (δD) was about 0.1% (<1%). The analytical details using the IRMS were presented elsewhere^39^.

The rain gauge data of an IMD station at Port Blair (11.67 °N, 92° 71’E) was provided by the National Data Center (NDC), IMD, Pune. The data consist of rainfall averaged over 24 hour. Other meteorological data, information on low pressure systems etc were also provided by the NDC and Weather Section of the IMD Pune. NOAA Interpolated Outgoing Longwave Radiation (OLR) data^42^ was used to quantify the convective activities. Additionally OLR data was used to show the convective anomalies graphically on pentad scale using a web-tool (http://iridl.ldeo.columbia.edu/). Daily gridded rainfall data of the Tropical Rain Measuring Mission (TRMM 3b42 v2; 0.25 × 0.25)^43^ was used to investigate the response of rainwater δ^18^O to rainfall over a wider spatial scale. Horizontal moisture flux vector [Q = (qu, qv)^44^, where q was the specific humidity (g kg^−1^) and u,v being the zonal and meridional wind vector (ms^−1^)] was calculated using the atmospheric circulation fields obtained from ERA-Interim^45^. Low level moisture convergence defined as ∇·(**V***q*) s^−1^ (ref. 46) [where **V** is the velocity vector (ms^−1^) and q (g.kg^−1^) the specific humidity] was also calculated.

In order to investigate the spectral characteristics of rainwater δ^18^O, daily data is required which is obviously not available. So the missing δ^18^O values were estimated by interpolating the original δ^18^O time series using cubic spline interpolation scheme. The interpolated values were constrained by maintaining the slope and intercept of the post interpolated local meteoric line (LML) the same as those of the pre-interpolated LML within the limits of uncertainty level. Additionally, the correlation coefficient of the δ^18^O vs. δD regression line after interpolation was maintained the same to that of the δ^18^O -δD regression line of the original data (i.e., before interpolation). In this context it may be mentioned that the interpolation of precipitation isotope data has been successfully used on spatial scale^47^ which we do here on temporal scale.

## Additional Information

**How to cite this article**: Chakraborty, S. *et al.* Atmospheric controls on the precipitation isotopes over the Andaman Islands, Bay of Bengal. *Sci. Rep.* **6**, 19555; doi: 10.1038/srep19555 (2016).

## Supplementary Material

Supplementary Information
