# Supplementary material for: Atmospheric controls on the precipitation isotopes over the Andaman Islands, Bay of Bengal
Source: Sci Rep. 2016 Jan 25;6:19555. doi: 10.1038/srep19555 (PMC4726406; doi:10.1038/srep19555)
Supplement: Supplementary Information [file srep19555-s1.pdf]

Supplementary Materials

Atmospheric controls on the precipitation isotopes over the Andaman Islands, Bay of Bengal

Authors: S. Chakraborty, N. Sinha, R. Chattopadhyay, S. Sengupta, P. M. Mohan, A. Datye

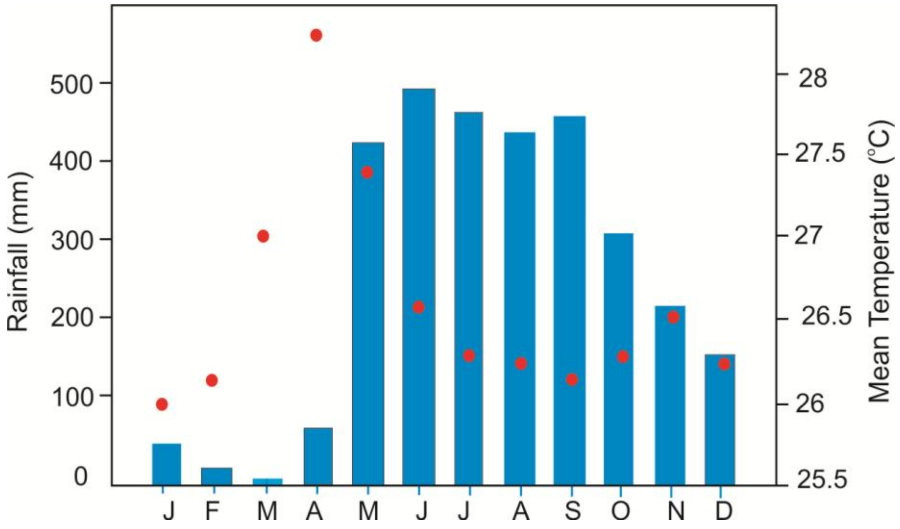

Figure S1 Climatological variation of rainfall (shaded bar) and average temperature (red circle) for Port Blair (based on the IMD data, shown in Laskar et al.[8]). Figure was created by licensed software Microsoft Excel and CorelDRAW.

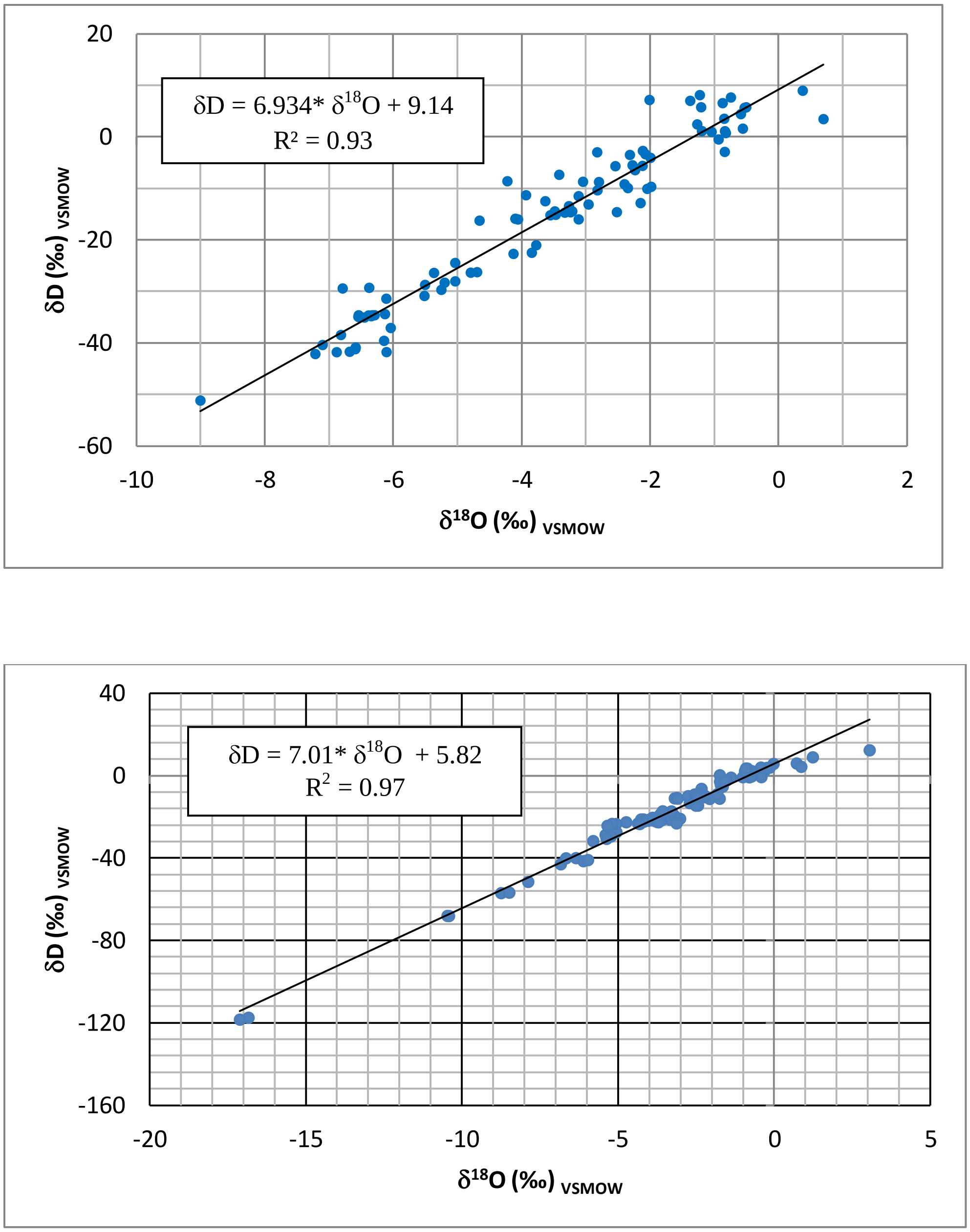

Figure S2 Local meteoric water line for the Andaman Islands for the year 2012 (upper panel) and 2013 (lower panel). The equation of the lines and the correlation coefficients are shown in the figure. Figures were made by licensed software Microsoft Office – Excel.

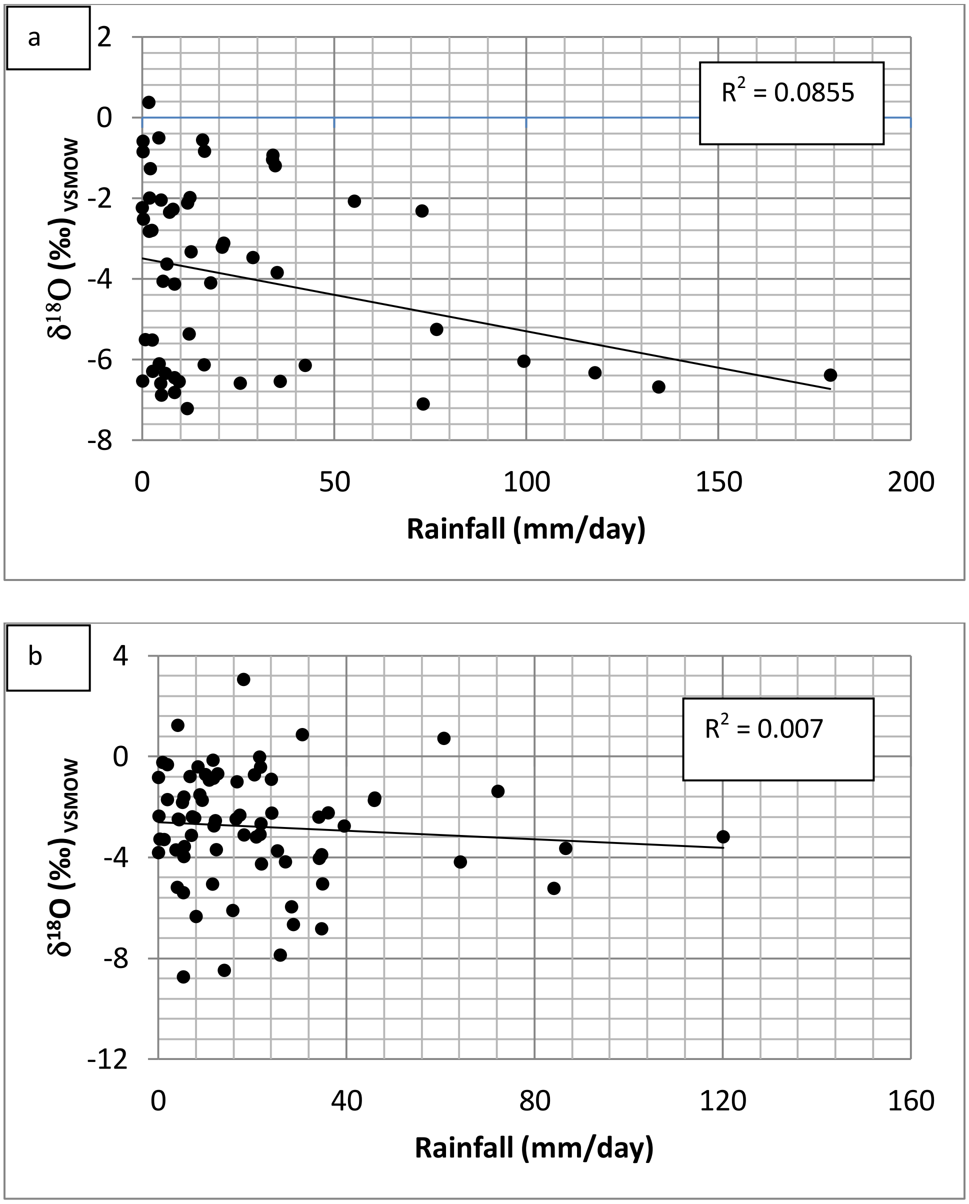

Figure-S3. Daily $\delta^{18}O$ data (JJAS) plotted as a function of rainfall amount (raingauge data) at Port Blair. Upper panel (a) is for the year 2012 and the lower panel(b) represents 2013. A weak amount effect is evident in case of year 2012 but the year 2013 practically shows no such effect. Figures were made by licensed software Microsoft Office – Excel.

Normalized d_excess (<-1)	Normalized d_excess (>+1)
1, May-2012	13-14, Jan-2012
4, 6, Jun-2012	2, Feb-2012
19, 25, 27, 29, Jul-2012	11, Apr-2012
11, 22, 30, Sept-2012	2, 4, 5, 9, 17, May-2012
1, 2, 17, Oct-2012	
	14, 16, July-2013
11, 23, 26, May-2013	13, 20, 29, 30, Sept-2013
12, 21, 24, Jun-2013	4, 5 , 9, Oct-2013
25, Jul-2013	13, 23-25, Nov-2013
2, 5, 10, Aug-2013	

Calculation:

$$\text{Normalized d\_excess} = (actual\ value - Mean)/Standard\ deviation$$

Table ST1: the table shows two sets of dates in which normalized d_excess < -1 (left column) and normalized d_excess >+1 (right column), based on which moisture convergence has been calculated and plotted in Figure 4. In order to demonstrate that origin of moisture is significantly different in these two sets we have taken three dates during monsoon and another three dates during non-monsoon season. 98-hr back trajectories have been calculated for these two sets of dates. Back Trajectories [produced by Hysplit[48] [http://ready.arl.noaa.gov/HYSPLIT.php, accessed on 24/05/2015] shown (Figure ST1) in left (right) column belong to the monsoon (non-monsoon) period when d_excess <-1 (>+1). The figure clearly shows that moistures having low d_excess are usually generated in equatorial Indian Ocean region whereas moisture with higher d_excess are typically generated in other areas such as, continental region.

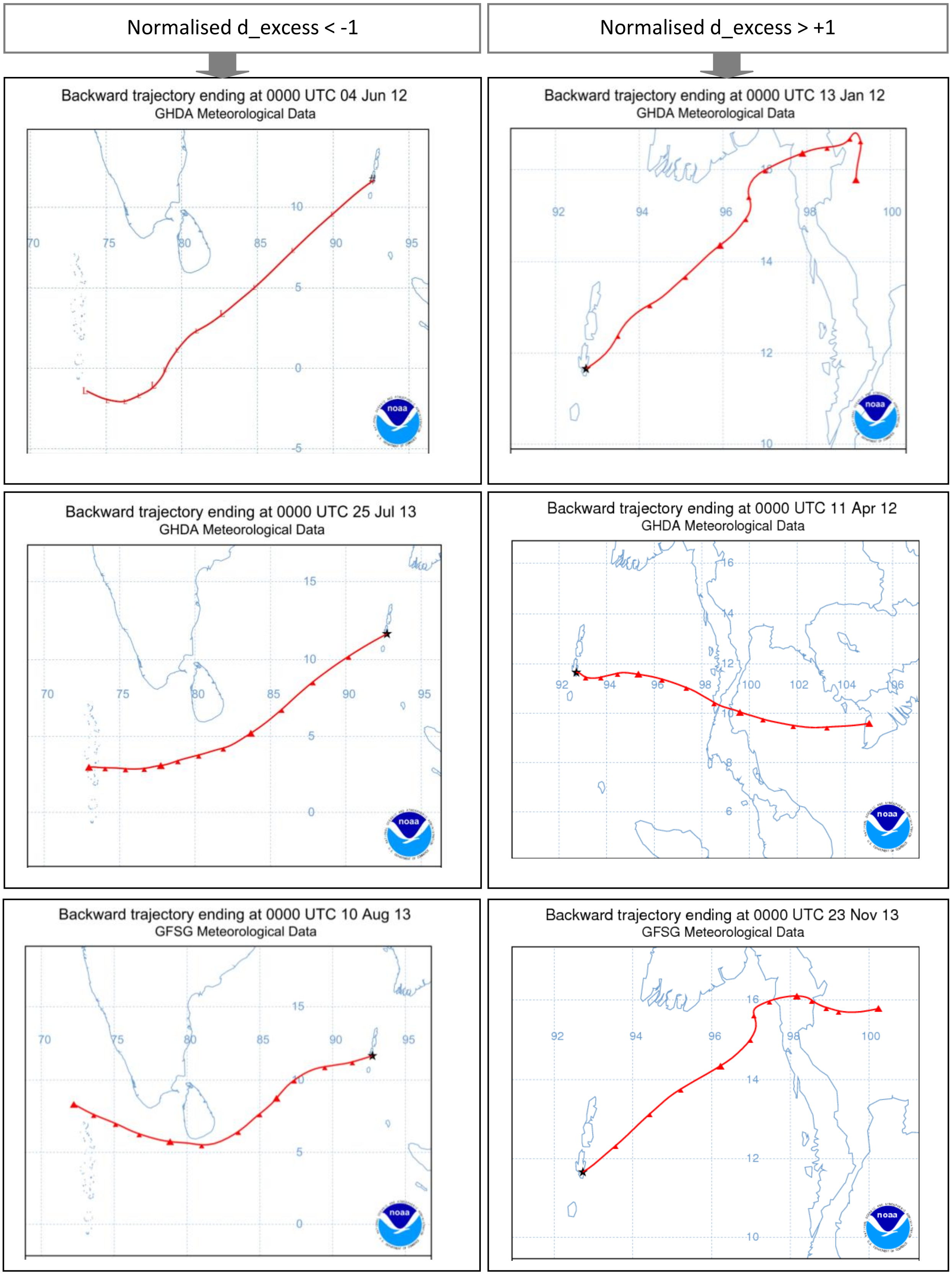

Figure ST1: Explanation has been provided in earlier caption (Table ST1).

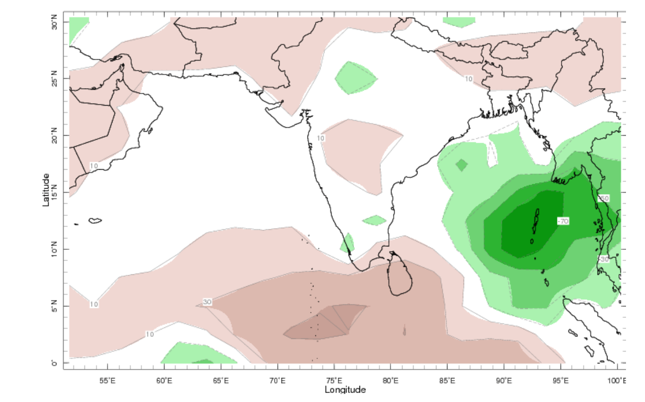

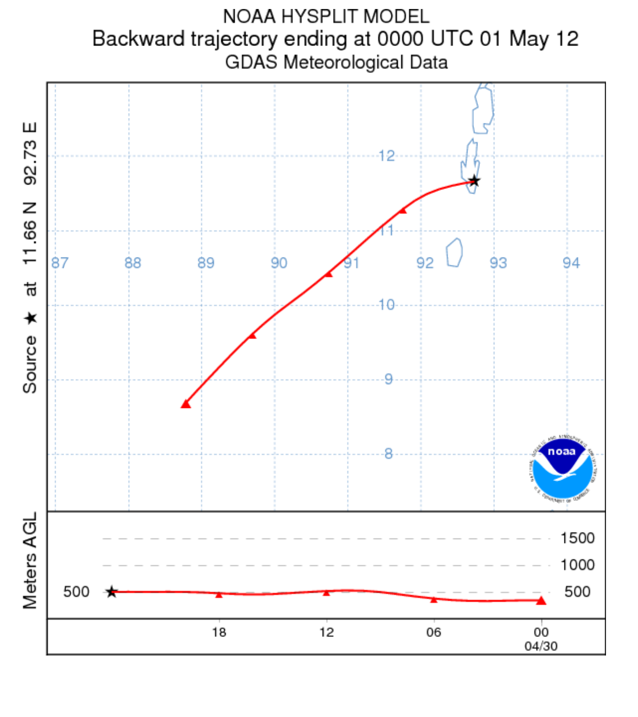

Figure S4 (top) Pentad OLR anomaly for 1-5 May 2012 showing deep convection in the north Indian Ocean region. Map was created using a webtool (http://iridl.ldeo.columbia.edu/). Green represents negative anomaly. Right- 24hr wind back trajectory plot for May 1-7, 2012, produced by Hysplit[48] (Draxler and Roplph 2003: http://ready.arl.noaa.gov/HYSPLIT.php, accessed on 01/12/2014) showing the air movement from the southern portion of the Bay of Bengal to the sample site (Port Blair, indicated as star). Latitudes and longitudes are marked on the grids. The bottom panel shows the temporal variation of the heights of the air masses in meters

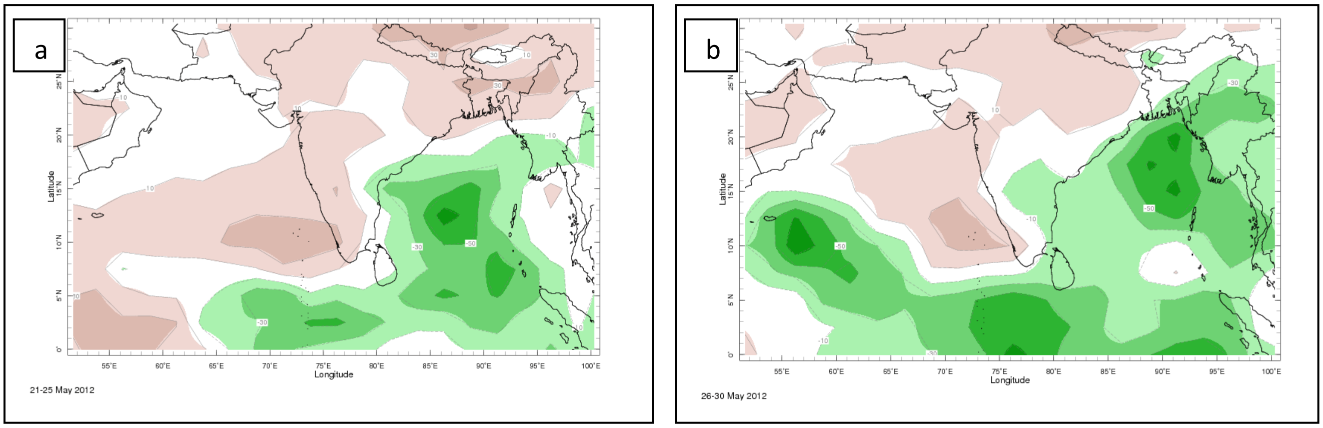

Figure S5 Pentad OLR anomaly for 21-25 May (a) and 26-30 May 2012 (b). Maps were created using a webtool (http://iridl.ldeo.columbia.edu/, accessed on 15/10/2014).

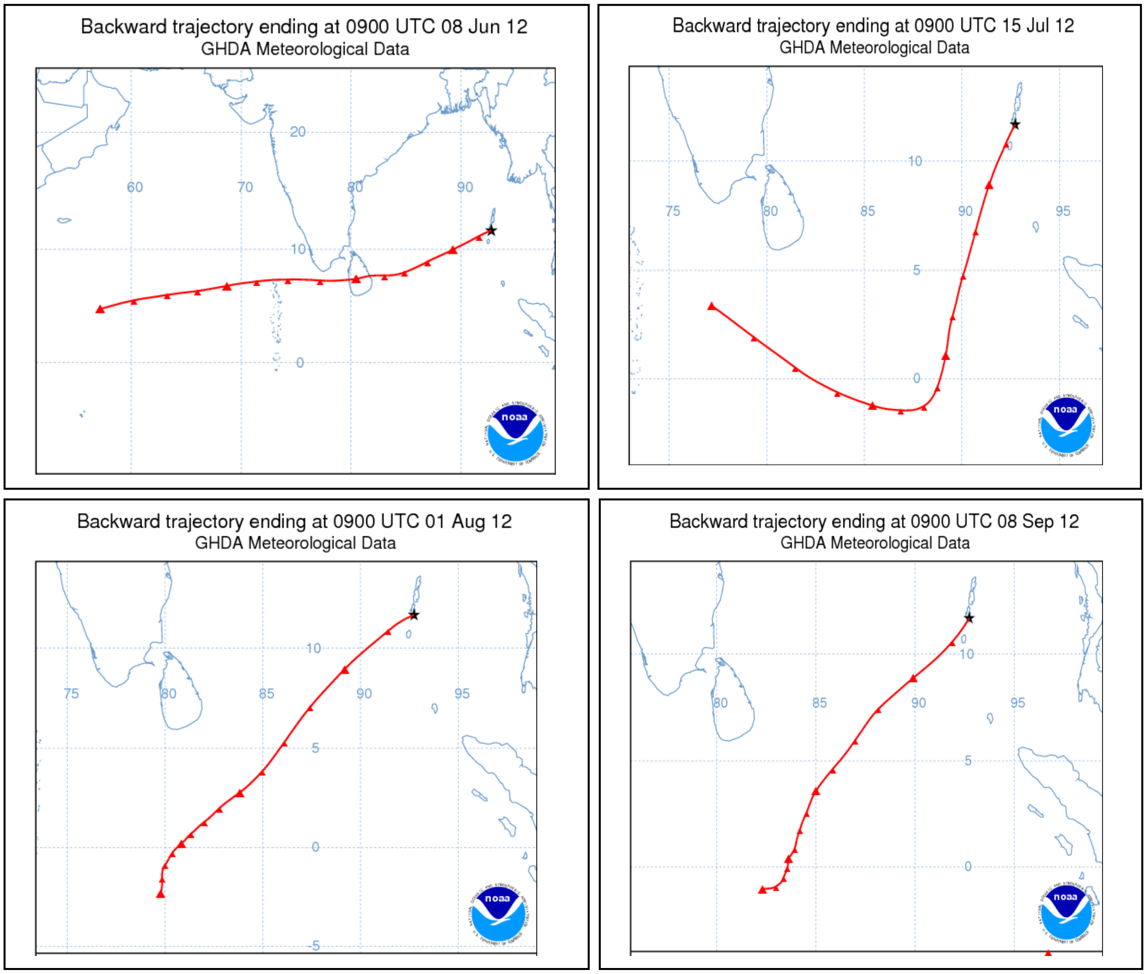

Figure S6 98 hr wind back trajectory plot for monsoon season (JJAS) showing the major moisture source at the sampling site is from the remote Indian Ocean. Trajectories were created using NOAA webtool (http://ready.arl.noaa.gov/HYSPLIT.php, accessed on 24/05/2015).

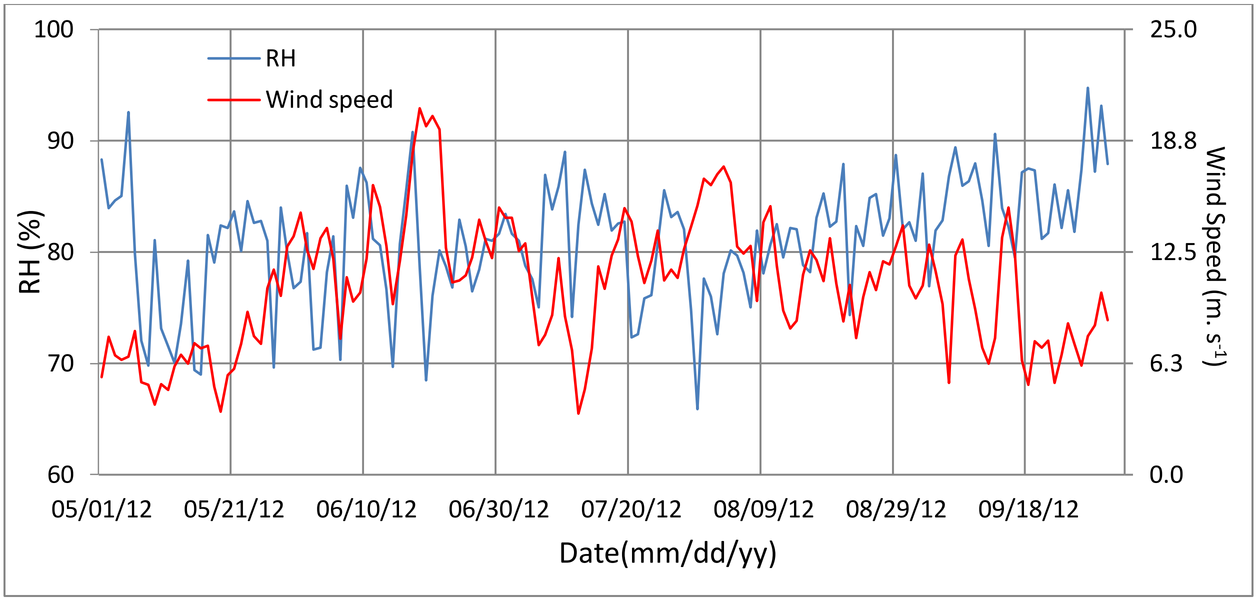

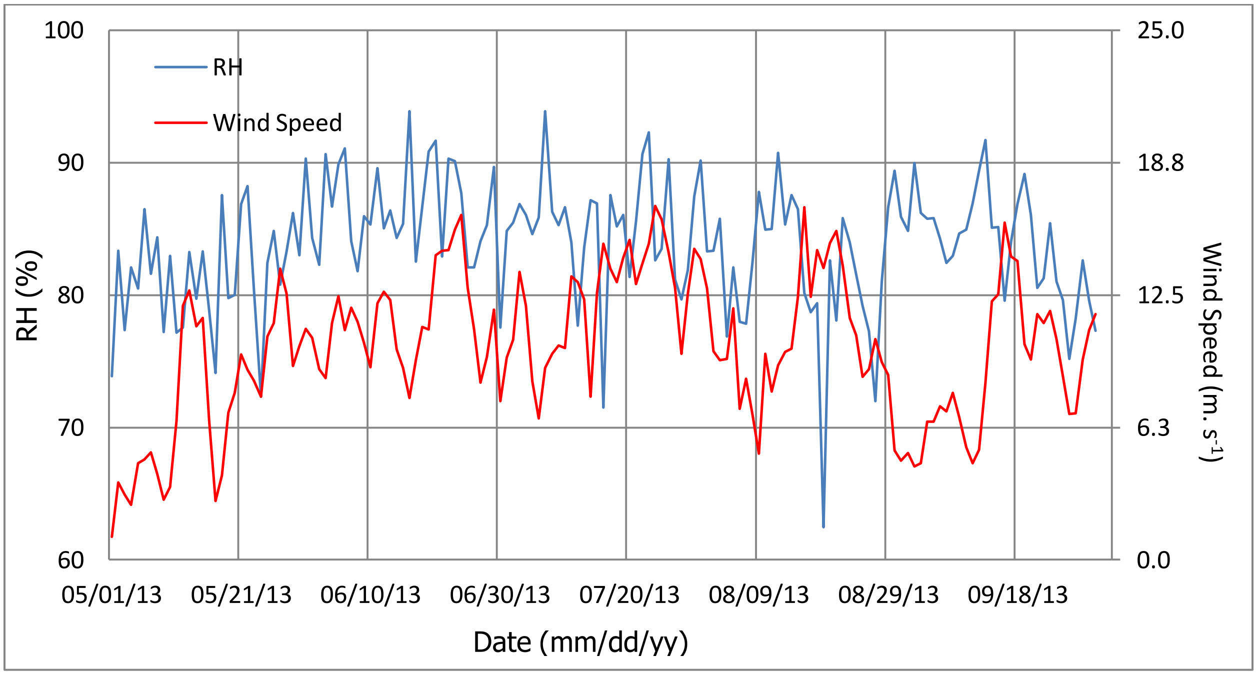

Figure S7 Relative humidity (in blue, left Y-axis) and Wind Speed (in red, right Y-axis) at 850 hPa on a 5x5 grid box over Port Blair. Data were obtained from ERA Interim (http://apps.ecmwf.int/; accessed on 24/05/2015). Upper panel is for the year 2012 and the lower panel is for the year 2013. Graphics produced using licensed version of MS Excel.

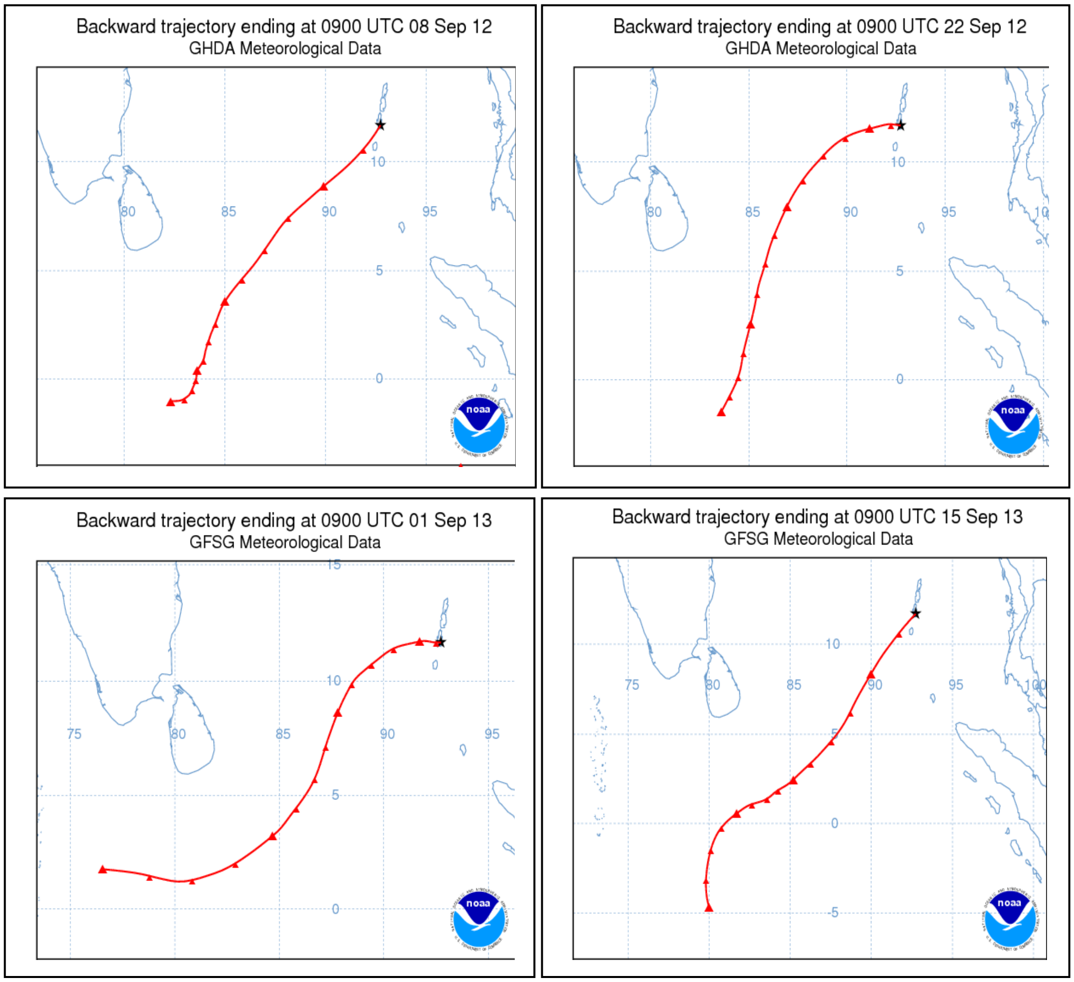

Figure S8 98 hr wind back trajectory plot for September month showing the major moisture source at the sampling site being the remote Indian Ocean in both the years. Trajectories were created using NOAA webtool (http://ready.arl.noaa.gov/HYSPLIT.php, accessed on 24/05/2015).
